# Incompatible quartets, triplets, and characters

**DOI:** 10.1186/1748-7188-8-11

**Published:** 2013-04-01

**Authors:** Brad Shutters, Sudheer Vakati, David Fernández-Baca

**Affiliations:** 1 Department of Computer Science, Iowa State University, Ames, IA 50011, USA

**Keywords:** Phylogenetics, Quartet compatibility, Triplet compatibility, Character compatibility, Perfect phylogeny

## Abstract

We study a long standing conjecture on the necessary and sufficient conditions for the compatibility of multi-state characters: There exists a function *f*(*r*) such that, for any set *C* of *r*-state characters, *C* is compatible if and only if every subset of *f*(*r*) characters of *C* is compatible. We show that for every *r*≥2, there exists an incompatible set *C* of *Ω*(*r*^2^)*r*-state characters such that every proper subset of *C* is compatible. This improves the previous lower bound of *f*(*r*)≥*r* given by Meacham (1983), and *f*(4)≥5 given by Habib and To (2011). For the case when *r*=3, Lam, Gusfield and Sridhar (2011) recently showed that *f*(3)=3. We give an independent proof of this result and completely characterize the sets of pairwise compatible 3-state characters by a single forbidden intersection pattern.

Our lower bound on *f*(*r*) is proven via a result on quartet compatibility that may be of independent interest: For every *n*≥4, there exists an incompatible set *Q* of *Ω*(*n*^2^) quartets over *n* labels such that every proper subset of *Q* is compatible. We show that such a set of quartets can have size at most 3 when *n*=5, and at most *O*(*n*^3^) for arbitrary *n*. We contrast our results on quartets with the case of rooted triplets: For every *n*≥3, if *R* is an incompatible set of more than *n*−1 triplets over *n* labels, then some proper subset of *R* is incompatible. We show this bound is tight by exhibiting, for every *n*≥3, a set of *n*−1 triplets over *n* taxa such that *R* is incompatible, but every proper subset of *R* is compatible.

## Background

The multi-state character compatibility (or perfect phylogeny) problem is a basic question in computational phylogenetics [1]. Given a set *C* of characters, we are asked whether there exists a phylogenetic tree that displays every character in *C*; if so, *C* is said to be compatible, and incompatible otherwise. The problem is known to be NP-complete [2,3], but certain special cases are known to be polynomially-solvable [4-10]. See [11] for more on the perfect phylogeny problem.

In this paper we study a long standing conjecture on the necessary and sufficient conditions for the compatibility of multi-state characters.

### Conjecture 1

There exists a function *f*(*r*) such that, for any set *C* of *r*-state characters, *C* is compatible if and only if every subset of *f*(*r*) characters of *C* is compatible.

If Conjecture 1 is true, it would follow that we can determine if any set *C* of *r*-state characters is compatible by testing the compatibility of each subset of *f*(*r*) characters of *C*, and, in case of incompatibility, output a subset of at most *f*(*r*) characters of *C* that is incompatible. This would allow us to reduce the character removal problem (i.e., finding a subset of characters to remove from *C* so that the remaining characters are compatible) to *f*(*r*)-hitting set which is fixed-parameter tractable [12].

A classic result on binary character compatibility shows that *f*(2)=2; see [1,6,13-15]. In 1975, Fitch [16,17] gave an example of a set *C* of three 3-state characters such that *C* is incompatible, but every pair of characters in *C* is compatible; showing that *f*(3)≥3. In 1983, Meacham [15] generalized this example to *r*-state characters for every *r*≥3 demonstrating a lower bound of *f*(*r*)≥*r* for all *r*; see also [9]. For the case of *r*=3, Lam, Gusfield, and Sridhar [9] recently established that *f*(3)=3.

While the previous results could lead one to conjecture that *f*(*r*)=*r* for all *r*, Habib and To [18] recently disproved this possibility by exhibiting a set *C* of five 4-state characters such that *C* is incompatible, but every proper subset of the characters in *C* are compatible, showing that *f*(4)≥5. They conjectured that *f*(*r*)≥*r*+1 for every *r*≥4.

The main result of this paper is to prove the conjecture stated in [18] by giving a quadratic lower bound on *f*(*r*). Formally, we show that for every *r*≥2, there exists a set *C* of *r*-state characters such that all of the following conditions hold. 

1. *C* is incompatible.

2. Every proper subset of *C* is compatible.

3. $|C|=\left\lfloor \frac{r}{2}\right\rfloor \cdot \left\lceil \frac{r}{2}\right\rceil +1$.

Therefore, $f(r)\geq \left\lfloor \frac{r}{2}\right\rfloor \cdot \left\lceil \frac{r}{2}\right\rceil +1$ for every *r*≥2.

Our proof relies on a new result on quartet compatibility we believe is of independent interest. We show that for every *n*≥4, there exists a set *Q* of quartets over a set of *n* labels such that all of the following conditions hold. 

1. *Q* is incompatible.

2. Every proper subset of *Q* is compatible.

3. $|Q|=\left\lfloor \frac{n-2}{2}\right\rfloor \cdot \left\lceil \frac{n-2}{2}\right\rceil +1$.

This is an improvement over the previous lower bound on the maximum cardinality of such an incompatible set of quartets of *n*−2 given in [3]. We show that such a set of quartets can have size at most 3 when *n*=5, and at most *O*(*n*^3^) for arbitrary *n*. We note here that the construction given in [18] showing that *f*(4)≥5 can be viewed as a special case of the construction given here when *n*=6.

We study the compatibility of three-state characters further. The work of [9] completely characterized the sets of pairwise compatible 3-state characters by the existence of one of four forbidden intersection patterns. An alternative characterization of this result was given in [10] and was partially derived using the results of [9]. In this paper, we give a proof that *f*(3)=3 that is independent of the results in [9], and we completely characterize the sets of pairwise compatible 3-state characters by a single forbidden intersection pattern.

We contrast our result on quartet compatibility with a result on the compatibility of rooted triplets: For every *n*≥3, if *R* is an incompatible set of triplets over *n* labels, and |*R*|>*n*−1, then some proper subset of *R* is incompatible. We show this bound is tight by exhibiting, for every *n*≥3, a set of *n*−1 triplets over *n* labels such that *R* is incompatible, but every proper subset of *R* is compatible.

## Preliminaries

Given a graph *G*, we represent the vertices and edges of *G* by *V*(*G*) and *E*(*G*) respectively. We use the abbreviated notation *uv* for an edge {*u*,*v*}∈*E*(*G*). For any *e*∈*E*(*G*), *G*−*e* represents the graph obtained from *G* by deleting edge *e*. For an integer *i*, we use [*i*] to represent the set {1,2,⋯,*i*}.

### Unrooted phylogenetic trees

**Figure 1:**
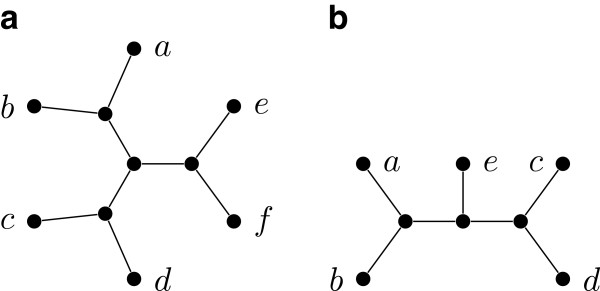
**A phylogenetic tree and a restricted subtree.** (**a**) shows a tree *T* witnessing that the quartets ***q***_**1**_=***ab|ce***, ***q***_**2**_=***cd|bf***, and ***q***_**3**_=***ad|ef*** are compatible; ***T*** is also a witness that the characters $\chi_{q_{1}}=\mathrm{ab}|\text{ce}|d|f$, $\chi_{q_{2}}=\mathrm{cd}|\text{bf}|a|e$, and $\chi_{q_{3}}=\mathrm{ad}|\text{ef}|b|c$ are compatible; (**b**) shows ***T|{a,b,c,d,e}***.

An *unrooted phylogenetic tree* (or just *tree*) is a tree *T* whose leaves are in one to one correspondence with a label set *L*(*T*), and has no vertex of degree two. See Figure 1(a) for an example. For a collection T of trees, the *label set* of T, denoted L(T), is the union of the label sets of the trees in T. A tree is *binary* if every internal (non-leaf) vertex has degree three. A *quartet* is a binary tree with exactly four leaves. A quartet with label set {*a*,*b*,*c*,*d*} is denoted *ab*|*cd* if the path between the leaves labeled *a* and *b* does not intersect with the path between the leaves labeled *c* and *d*.

For a tree *T*, and a label set *L*⊆*L*(*T*), the *restriction* of *T* to *L*, denoted by *T*|*L*, is the tree obtained from the minimal subtree of *T* connecting all the leaves with labels in *L* by suppressing vertices of degree two. See Figure 1(b) for an example. A tree *Tdisplays* another tree *T*^′^, if *T*^′^ can be obtained from *T*|*L*(*T*^′^) by contracting edges. A tree *T* displays a collection of trees T if *T* displays every tree in T. If such a tree *T* exists, then we say that T is *compatible*; otherwise, we say that T is *incompatible*. See Figure 1(a) for an example. Determining if a collection of unrooted trees is compatible is NP-complete [3].

### Multi-state characters

There is also a notion of compatibility for sets of partitions of a label set *L*. A *characterχ* on *L* is a partition of *L*; the parts of *χ* are called *states*. If *χ* has at most *r* parts, then *χ* is an *r*-state character. Given a tree *T* with *L*=*L*(*T*) and a state *s* of *χ*, we denote by *T*_*s*_(*χ*) the minimal subtree of *T* connecting all leaves with labels having state *s* for *χ*. We say that *χ* is *convex* on *T*, or equivalently *T displaysχ*, if the subtrees *T*_*i*_(*χ*) and *T*_*j*_(*χ*) are vertex disjoint for all states *i* and *j* of *χ* where *i*≠*j*. A collection *C* of characters is *compatible* if there exists a tree *T* on which every character in *C* is convex. If no such tree exists, then we say that *C* is *incompatible*. See Figure 1(a) for an example. The *perfect phylogeny problem* (or *character compatibility problem*) is to determine whether a given set of characters is compatible.

For a collection *C* of characters, the *intersection graph* of *C* which we will denote by *G*(*C*), is the undirected graph *G*=(*V*,*E*) which has a vertex *c*_*i*_ for each character *c*∈*C* and each state *i* of *c*, and an edge *c*_*i*_*d*_*j*_ precisely when there is a taxon having state *i* for character *c* and state *j* for character *d*. Note that *G*(*C*) cannot have an edge between vertices associated with different states of the same character.

A graph *G* is *chordal* if there are no induced chordless cycles of length four or greater in *H*. In [19], Buneman established a fundamental connection between the perfect phylogeny problem and chordal graphs which we now describe. For a given set *C* of characters, suppose we color each of the vertices of *G*(*C*) by assigning a unique color to each character *c*∈*C*, and giving each vertex of *G*(*C*) corresponding to a state of *c* with the color assigned to the character *c*. A *proper triangulation* of *G*(*C*) is a chordal supergraph of *G*(*C*) such that every edge has endpoints with different colors.

#### 

**Theorem 1. **A set *C* of characters is compatible if and only if *G*(*C*) has a proper triangulation.

Since there is no proper triangulation for a cycle in *G*(*C*) involving only vertices from two characters, we have the following corollary.

#### 

**Corollary 1.** Let *C* be a collection of two characters. Then *C* is compatible if and only if *G*(*C*) is acyclic.

### Quartet rules

We now introduce *quartet (closure) rules* which were originally used in the contexts of psychology [20] and linguistics [21]. The idea is that for a collection *Q* of quartets, any tree that displays *Q* may also necessarily display another quartet *q*∉*Q*, and if so we write *Q*⊩*q*.

#### 

**Example 1.** Let *Q*={*ab*|*ce*,*ae*|*cd*}. Then the tree of Figure 1(b) displays *Q*, and furthermore, it is easy to see that it is the only tree that displays *Q*. Hence, *Q*⊩*ab*|*de*, *Q*⊩*ab*|*cd*, and *Q*⊩*be*|*cd*.

We use the following quartet rules in this paper:

(R1) {ab|cd,ab|ce}⊩ab|de

(R2) {ab|cd,ac|de}⊩ab|ce

For the purposes of this paper, we define the *closure* of an arbitrary collection *Q* of quartets, denoted *Q*^∗^, as the minimal set of quartets that contains *Q*, and has the property that if for some *q*_1_,*q*_2_∈*Q*^∗^, {*q*_1_,*q*_2_}⊩*q*_3_ using either (R1) or (R2), then *q*_3_∈*Q*^∗^. Clearly, any tree that displays *Q* must also display *Q*^∗^. We will use the following lemma which follows by repeated application of (R!) and is formally proven in [22].

#### 

**Lemma 1.** Let *Q* be an arbitrary set of quartets with {*x*,*y*,*z*_1_,…,*z*_*k*_}⊆*L*(*Q*). If

$$\overset{k-1}{\underset{i=1}{⋃}}\{\text{xy}|z_{i}z_{i+1}\}\subseteq Q^{*}\;,$$

then *xy*|*z*_1_*z*_*k*_∈*Q*^∗^.

We refer the reader to [1,23] for more on quartet rules.

## Incompatible quartets

For every *s*,*t*≥2, we fix a set of labels *L*_*s*,*t*_={*a*_1_,*a*_2_,…,*a*_*s*_,*b*_1_,*b*_2_,…,*b*_*t*_} and define the set

$$Q_{s,t}=\{a_{1}b_{1}|a_{s}b_{t}\}\cup \overset{s-1}{\underset{i=1}{⋃}}\overset{t-1}{\underset{j=1}{⋃}}\{a_{i}a_{i+1}|b_{j}b_{j+1}\}$$

of quartets with *L*(*Q*_*s*,*t*_)=*L*_*s*,*t*_. We denote the quartet *a*_1_*b*_1_|*a*_*s*_*b*_*t*_ by *q*_0_, and a quartet of the form *a*_*i*_*a*_*i*+1_|*b*_*j*_*b*_*j*+1_ by *q*_*i*,*j*_.

### 

**Observation 1. **For all *s*,*t* ≥ 2, |*Q*_*s*,*t*_|=(*s*−1)(*t*−1) + 1.

### 

**Lemma 2. **For all *s*,*t*≥2, *Q*_*s*,*t*_ is incompatible.

### 

**Proof.** For each *i*∈[*s*−1],

$$\overset{t-1}{\underset{j=1}{⋃}}\{a_{i}a_{i+1}|b_{j}b_{j+1}\}\subseteq Q_{s,t}\subseteq Q_{s,t}^{*}.$$

Then, by Lemma 1, it follows that for each *i*∈[*s*−1], $a_{i}a_{i+1}|b_{1}b_{t}\in Q_{s,t}^{*}$. So,

$$\overset{s-1}{\underset{i=1}{⋃}}\{b_{1}b_{t}|a_{i}a_{i+1}\}\subseteq Q_{s,t}^{*}.$$

Then, again by Lemma 1, it follows that $b_{1}b_{t}|a_{1}a_{s}\in Q_{s,t}^{*}$. But then $\{a_{1}b_{1}|a_{s}b_{t},b_{1}b_{t}|a_{1}a_{s}\}\subseteq Q_{s,t}^{*}$. It follows that any tree that displays *Q*_*s*,*t*_ must display both *a*_1_*b*_1_|*a*_*s*_*b*_*t*_ and *b*_1_*b*_*t*_|*a*_1_*a*_*s*_. However, no such tree exists. Hence, *Q*_*s*,*t*_ is incompatible. □

### 

**Lemma 3.** For all *s*,*t*≥2, every proper subset of *Q*_*s*,*t*_ is compatible.

### 

**Proof.** Since every subset of a compatible set of quartets is compatible, it suffices to show that for every *q*∈*Q*_*s*,*t*_, *Q*_*s*,*t*_∖{*q*} is compatible. Let *q*∈*Q*_*s*,*t*_. Either *q*=*q*_0_ or *q*=*q*_*x*,*y*_ for some 1≤*x*<*s* and 1≤*y*<*t*. In either case, we exhibit a tree witnessing that *Q*_*s*,*t*_∖{*q*} is compatible. □

**Figure 2:**
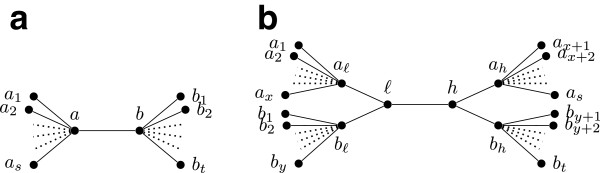
**Illustrating the proof of Lemma 3.** (**a**) Case 1: a tree that displays *Q*_*s*,*t*_∖{*q*_0_}. (**b**) Case 2: a tree that displays *Q*_*s*,*t*_∖{*q*_*x*,*y*_}.

•*Case 1*. Suppose *q*=*q*_0_. We build the tree *T* as follows: There is a node *ℓ* for each label *ℓ*∈*L*_*s*,*t*_ and two additional nodes *a* and *b* along with the edge *ab*. There is an edge *a*_*x*_*a* for every *a*_*x*_∈*L*_*s*,*t*_, and an edge *b*_*x*_*b* for every *b*_*x*_∈*L*_*s*,*t*_. There are no other nodes or edges in *T*. See Figure 2(a) for an illustration. Now consider any quartet *q*∈*Q*_*s*,*t*_∖{*q*_0_}. Then *q*=*a*_*i*_*a*_*i*+1_|*b*_*j*_*b*_*j*+1_ for some 1≤*i*<*s* and 1≤*j*<*t*. Then, the minimal subgraph of *T* connecting leaves with labels in {*a*_*i*_,*a*_*i*+1_,*b*_*j*_,*b*_*j*+1_} is the quartet *q*. Hence *T* displays *q*.

•*Case 2*. Suppose *q*=*q*_*x*,*y*_ for some 1≤*x*<*s* and 1≤*y*<*t*. We build the tree *T* as follows: There is a node *ℓ* for each label *ℓ*∈*L*_*s*,*t*_ and six additional nodes *a*_*ℓ*_, *b*_*ℓ*_, *ℓ*, *h*, *a*_*h*_, and *b*_*h*_. There are edges *a*_*ℓ*_*ℓ*, *b*_*ℓ*_*ℓ*, *ℓh*, *ha*_*h*_, and *hb*_*h*_. For every *a*_*i*_∈*L*_*s*,*t*_, there is an edge *a*_*i*_*a*_*ℓ*_ if *i*≤*x*, and an edge *a*_*i*_*a*_*h*_ if *i*>*x*. For every *b*_*j*_∈*L*_*s*,*t*_ there is an edge *b*_*j*_*b*_*ℓ*_ if *j*≤*x*, and an edge *b*_*j*_*b*_*h*_ if *j*>*y*. There are no other nodes or edges in *T*. See Figure 2(b). Now consider any quartet *q*∈*Q*_*s*,*t*_∖{*q*_*x*,*y*_}. Either *q*=*q*_0_ or *q*=*q*_*i*,*j*_ where *i*≠*x* or *j*≠*y*. If *q*=*q*_0_, then the minimal subgraph of *T* connecting leaves with labels in {*a*_1_,*b*_1_,*a*_*s*_,*b*_*t*_} is the subtree of *T* induced by the nodes in {*a*_1_,*a*_*ℓ*_,*ℓ*,*b*_*ℓ*_,*b*_1_,*a*_*s*_,*a*_*h*_,*h*,*b*_*h*_,*b*_*t*_}. Suppressing all degree two vertices results in a tree that is the same as *q*_0_. So *T* displays *q*. So assume that *q*=*a*_*i*_*a*_*i*+1_|*b*_*j*_*b*_*j*+1_ where *i*≠*x* or *j*≠*y*. We define the following subset of the nodes in *T*:

$$\;V\;=\;\left\{\begin{array}{c} \;\{a_{i},a_{i+1},a_{\ell },\ell ,b_{\ell },b_{j},b_{j+1}\}\;\;\;\text{if}\;i<x\;\text{and}\;j<y,\\ \;\{a_{i},a_{i+1},a_{\ell },\ell ,b_{y},b_{\ell },h,b_{h},b_{y+1}\}\;\;\;\;\;\text{if}\;i<x\;\text{and}\;j=y,\\ \;\{a_{i},a_{i+1},a_{\ell },\ell ,h,b_{h},b_{j},b_{j+1}\}\;\;\;\text{if}\;i<x\;\text{and}\;j>y,\\ \;\{a_{x},a_{\ell },\ell ,h,a_{h},a_{x+1},b_{\ell },b_{j},b_{j+1}\}\;\;\;\;\;\text{if}\;i=x\;\text{and}\;j<y,\\ \;\{a_{x},a_{\ell },\ell ,h,a_{h},a_{x+1},b_{h},b_{j},b_{j+1}\}\;\;\;\;\;\text{if}\;i=x\;\text{and}\;j>y,\\ \;\{a_{j},a_{j+1},a_{h},h,\ell ,b_{\ell },b_{j},b_{j+1}\}\;\;\;\;\text{if}\;i>x\;\text{and}\;j<y,\\ \;\{a_{j},a_{j+1},a_{h},h,b_{y},b_{\ell },\ell ,b_{h},b_{y+1}\}\;\;\;\;\text{if}\;i>x\;\text{and}\;j=y,\\ \;\{a_{j},a_{j+1},a_{h},h,b_{h},b_{j},b_{j+1}\}\;\;\;\text{if}\;i>x\;\text{and}\;j>\mathrm{y.} \end{array}\right)$$

Now, the subgraph of *T* induced by the nodes in *V* is the minimal subgraph of *T* connecting leaves with labels in *q*. Suppressing all degree two vertices gives *q*. Hence, *T* displays *q*.

With $s=\left\lfloor \frac{n}{2}\right\rfloor$ and $t=\left\lceil \frac{n}{2}\right\rceil$, Observation 1and Lemmas 2 and 3 imply the following theorem.

### 

**Theorem 2.** For every integer *n*≥4, there exists a set *Q* of quartets over *n* taxa such that all of the following conditions hold. 

1. *Q* is incompatible.

2. Every proper subset of *Q* is compatible.

3. $|Q|=\left\lfloor \frac{n-2}{2}\right\rfloor \cdot \left\lceil \frac{n-2}{2}\right\rceil +1$.

### Incompatible quartets on five taxa

When *Q* is a set of quartets over five taxa, we show that the set of quartets given by Theorem 2 is as large as possible. We hope that the technique used in the proof of the following theorem might be useful in proving tight bounds for *n*>5.

#### 

**Theorem 3.** If *Q* is an incompatible set of quartets over five taxa such that every proper subset of *Q* is compatible, then |*Q*|≤3.

#### 

**Proof.** Let *Q* be an incompatible set of quartets with *L*(*Q*)={*a*,*b*,*c*,*d*,*e*} and *q*_0_=*ab*|*cd*∈*Q*. We will show that *Q* contains an incompatible subset of at most three quartets. If *Q* contains two different quartets on the same four taxa, then *Q* must contain an incompatible pair of quartets. So, we may assume that each quartet is on a unique subset of four of the five taxa. Hence, every pair of quartets in *Q* shares three taxa in common. We have the following two cases. 

•*Case 1*: *Q* contains at least one of the quartets *ac*|*be*, *ac*|*de*, *ad*|*be*, *ad*|*ce*, *ae*|*bc*, *ae*|*bd*, *bc*|*de*, or *bd*|*ce*. W.l.o.g. we may assume that *Q* contains *q*_1_=*ac*|*de*, as all other cases are symmetric. By (R2), {*q*_0_,*q*_1_}⊩*ab*|*ce*. Then, by (R1), {*q*_0_,*q*_1_,*ab*|*ce*}⊩*ab*|*de*. Then, again by (R1), {*q*_0_,*q*_1_,*ab*|*ce*,*ab*|*de*}⊩*bc*|*de*. Now let *Q*^′^={*q*_0_,*q*_1_,*ab*|*ce*,*ab*|*de*,*bc*|*de*}. Now, any quartet in *Q* must be either in *Q*^′^ or be pairwise incompatible with a quartet in *Q*^′^. Since *Q*^′^ is compatible, but by assumption, *Q* is incompatible, *Q* must contain a quartet *q*_2_ that is pairwise incompatible with some quartet in *Q*^′^. Hence, {*q*_0_,*q*_1_,*q*_2_} is an incompatible subset of *Q*.

•*Case 2*: *Q* contains none of the quartets *ac*|*be*, *ac*|*de*, *ad*|*be*, *ad*|*ce*, *ae*|*bc*, *ae*|*bd*, *bc*|*de*, or *bd*|*ce*. Then every quartet in *Q* is either of the form *ab*|*xy* where {*x*,*y*}≠{*c*,*d*}, or *cd*|*xy* where {*x*,*y*}≠{*a*,*b*}. But then *Q* is compatible, contradicting our assumption that *Q* is incompatible.

In either case, the theorem holds. □

### Incompatible quartets on arbitrarily many taxa

We say a set *Q* of compatible quartets is *redundant* if for some *q*∈*Q*, *Q*∖{*q*}⊩*q*; otherwise, we say that *Q* is *irredundant*. The following lemma establishes a connection between sets of irredundant quartets and minimal sets of incompatible quartets.

#### 

**Lemma 4.** If *Q* is incompatible, but every proper subset of *Q* is compatible, then every proper subset of *Q* is irredundant.

#### 

**Proof. **Suppose that *Q* is incompatible and every proper subset of *Q* is compatible. Furthermore, suppose that some proper subset *Q*^′^ of *Q* is redundant. Since every compatible superset of a redundant set of quartets is also redundant, we may assume w.l.o.g., that there is a unique quartet *q*∈*Q*∖*Q*^′^ (i.e., |*Q*|=|*Q*^′^|+1). Since *Q*^′^ is redundant, there exists a *q*^′^∈*Q*^′^ such that *Q*^′^∖{*q*^′^}⊩*q*^′^. But then (*Q*^′^∖{*q*^′^})∪{*q*} is incompatible, contradicting that every proper subset of *Q* is compatible. □

It follows from Lemma 4 that any upper bound on the maximum cardinality of an irredundant set of quartets can be used to place an upper bound on the maximum cardinality of a set of quartets satisfying the first two conditions of Theorem 2. The theorem follows from [22].

#### 

**Theorem 4.** Let *Q* be a set of quartets over a set of *n* taxa. If *Q* is irredundant, then *Q* has cardinality at most (*n*−3)(*n*−2)^2^/3.

Lemma 4 together with Theorem 4 gives the following upper bound on the maximum cardinality of a set *Q* of quartets over *n*>5 taxa that satisfies the first two conditions of Theorem 2.

#### 

**Theorem 5.** Let *Q* be a set of incompatible quartets over a set of *n* taxa such that every proper subset of *Q* is compatible. Then |*Q*|≤(*n*−3)(*n*−2)^2^/3+1.

## Incompatible characters

There is a natural correspondence between quartet compatibility and character compatibility that we now describe. Let *Q* be a set of quartets, *n*=|*L*(*Q*)|, and *r*=*n*−2. For each *q*=*ab*|*cd*∈*Q*, we define the *r*-state character corresponding to *q*, denoted *χ*_*q*_, as the character where *a* and *b* have state 0 for *χ*_*q*_; *c* and *d* have state 1 for *χ*_*q*_; and, for each *ℓ*∈*L*(*Q*)∖{*a*,*b*,*c*,*d*}, there is a state *s* of *χ*_*q*_ such that *ℓ* is the only label with state *s* for character *χ*_*q*_ (see Example 2). We define the set of *r*-state characters corresponding to *Q* by

$$C_{Q}={⋃}_{q\in Q}\{\chi_{q}\}.$$

### 

**Example 2.** Consider the quartets and characters given in Figure 1(a): $\chi_{q_{1}}$ is the character corresponding to *q*_1_, $\chi_{q_{2}}$ is the character corresponding to *q*_2_, and $\chi_{q_{3}}$ is the character corresponding to *q*_3_.

The following lemma relating quartet compatibility to character compatibility is well known [24], and its proof is omitted here.

### 

**Lemma 5.** A set *Q* of quartets is compatible if and only if *C*_*Q*_ is compatible.

The next theorem allows us to use our result on quartet compatibility to establish a lower bound on *f*(*r*).

### 

**Theorem 6.** Let *Q* be a set of incompatible quartets over *n* labels such that every proper subset of *Q* is compatible, and let *r*=*n*−2. Then, there exists a set *C* of |*Q*|*r*-state characters such that *C* is incompatible, but every proper subset of *C* is compatible.

### 

**Proof.** We claim that *C*_*Q*_ is such a set of incompatible *r*-state characters. Since for two quartets *q*_1_,*q*_2_∈*Q*, $\chi_{q_{1}}\neq \chi_{q_{2}}$, it follows that |*C*_*Q*_|=|*Q*|. Since *Q* is incompatible, it follows by Lemma 5 that *C*_*Q*_ is incompatible. Let *C*^′^ be any proper subset of *C*. Then, there is a proper subset *Q*^′^ of *Q* such that $C^{\prime }=C_{Q^{\prime }}$. Then, since *Q*^′^ is compatible, it follows by Lemma 5 that *C*^′^ is compatible. □

Theorem 2 together with Theorem 6 gives the main theorem of this paper.

### 

**Theorem 7.** For every integer *r*≥2, there exists a set *C* of *r*-state characters such that all of the following hold. 

1. *C* is incompatible.

2. Every proper subset of *C* is compatible.

3. $|C|=\left\lfloor \frac{r}{2}\right\rfloor \cdot \left\lceil \frac{r}{2}\right\rceil +1$.

### 

**Proof.** By Theorem 2 and Observation 1, there exists a set *Q* of $\left\lfloor \frac{r}{2}\right\rfloor \cdot \left\lceil \frac{r}{2}\right\rceil +1$ quartets over *r*+2 labels that that are incompatible, but every proper subset is compatible, namely $Q_{\left\lfloor \frac{r+2}{2}\right\rfloor ,\left\lceil \frac{r+2}{2}\right\rceil }$. The theorem follows from Theorem 6. □

The quadratic lower bound on *f*(*r*) follows from Theorem 7.

### 

Corollary 2.

$$f(r)\geq \left\lfloor \frac{r}{2}\right\rfloor \cdot \left\lceil \frac{r}{2}\right\rceil +1$$.

### Three-State Characters

In the remainder of this section we focus on the case when *r*=3, and thus, fix *C* to be an arbitrary set of 3-state characters over a set *S* of taxa. Lam, Gusfield, and Sridhar [9] recently established that *f*(3)=3, and they completely characterized the sets of pairwise compatible 3-state characters by the existence of one of four forbidden intersection patterns. We give an independent proof that *f*(3)=3. We then completely characterize the sets of pairwise compatible 3-state characters by a single forbidden intersection pattern. Our proof uses several structural results from the algorithm for the three-state perfect phylogeny problem given by Kannan and Warnow [7].

#### The Algorithm of Kannan and Warnow

The algorithm of [7] takes a divide and conquer approach to determining the compatibility of a set of three-state characters. An instance is reduced to subproblems by finding a partition *S*_1_,*S*_2_ of the taxon set *S* of *C* with both of the following properties: 

1. 2≤|*S*_*i*_|≤*n*−2,*i*=1,2.

2. Whenever *C* is compatible *S* there is a perfect phylogeny *P* that contains an edge *e* whose removal breaks *P* into subtrees *P*_1_ and *P*_2_ with *L*(*P*_*i*_)=*S*_*i*_,*i*=1,2.

A partition of *S* satisfying both of these properties is a *legal partition*, and the following theorem shows that finding such a partition for a given set of characters is the crux of the algorithm.

##### 

**Theorem 8.**[7] Given a set *C* of three state characters, we can in *O*(*nk*) time either find a legal partition of *S* of determine that the set of characters is incompatible.

#### Finding a legal partition

We now discuss the manner in which such a legal partition is found for a set of three-state characters *C*. Let *T* be a tree witnessing that *C* is compatible. The *canonical labeling* of *T* is the labeling where, for each internal node *v* of *T*, and each character *α*∈*C*, if there are leaves *x* and *y* in different components of *T*−{*v*} such that *α*(*x*)=*α*(*y*), then *α*(*v*)=*α*(*x*); otherwise *α*(*v*)=∗ where ∗ denotes a *dummy* state for *C*. Note that such a labeling of *T* always exists and is unique. We will assume that every compatible tree for *C* is canonically labeled.

**Figure 3:**
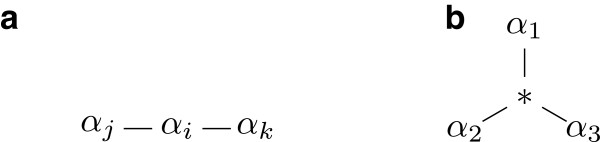
**The four possible realizable tree-structures for a three-state character*α*.** (**a**) A path P^*i*^ for each *i* ∈ {1,2,3}. (**b**) A star S^∗^.

The *tree-structure* for a character *α* in *T* is formed by repeatedly contracting edges of *T* connecting nodes that have the same state (other than ∗) for *α*. Note that this tree does not depend on the sequence of edge-contractions and is thus well defined. Furthermore, there is exactly one node for each state (other than the dummy state) of *α*, and each node labeled by ∗ has degree at least three. A tree-structure for *α* that is formed from some compatible tree for *C* is called a *realizable tree-structure* for *α*. There are four possible realizable tree-structures for a three-state character *α* which are shown in Figure 3.

To find a realizable tree structure for a character *α*, the algorithm examines the pairwise intersection patterns of *α* with every other character *β∈*∈c*C*, and applies the following rules to rule out possible tree structures for *α*.

##### 

**Rule 1.** Let *α* and *β* be two characters of *C*. If, under some relabeling of the states of *α* and *β*, we have that *α*_1_⊆*β*_1_, *α*_2_∩*β*_2_≠*∅*, and *α*_3_∩*β*_2_≠*∅*, then *P*^1^ is not a realizable tree-structure for *α*. If this is the case, we say that *α* and *β* match Rule 1 with respect to *α*_1_.

##### 

**Rule 2.**^a^Let *α* and *β* be two characters of *C*. If, under some relabeling of the states of *α* and *β*, we have that *α*_1_∩*β*_1_≠*∅*, *α*_2_∩*β*_1_≠*∅*, *α*_2_∩*β*_2_≠*∅*, and *α*_3_∩*β*_2_≠*∅*, then *P*^2^ is the only possible realizable tree-structure for *α*. If this is the case, we say that *α* and *β* match Rule 2 with respect to *α*_2_.

The set $Q_{\alpha }^{C}$ of *candidate* tree-structures for *α* are all of those possible tree-structures for *α* that are not ruled out after comparing the intersection pattern of *α* with every other character in *C* and applying Rules 1 and 2.

The following theorem which follows from [7] shows that a legal partition is found by choosing an arbitrary *α*∈*C* for which $Q_{\alpha }^{C}\neq \emptyset$. Furthermore, if there is an *α*∈*C* for which $Q_{\alpha }^{C}=\emptyset$, then *C* is incompatible.

##### 

**Theorem 9** ( [7]).If $Q_{\alpha }^{C}\neq \emptyset$, then we can find a legal partition of *S*.

##### 

**Corollary 3.** A set *C* of 3-state characters is compatible if and only if $Q_{\alpha }^{C}\neq \emptyset$ for every *α*∈*C*.

#### Tight bounds on three-state character compatibility

We use Corollary 3 to give upper bounds on the maximum cardinality of a minimal set of incompatible three-state characters.

##### 

**Theorem 10.**Let *C* be a set of three-state characters on species set *S*. Then *C* is incompatible if and only if there exists a character *α*∈*C*, and two distinct states *α*_*i*_ and *α*_*j*_ of *α*, such that both of the following hold: 

1. There is a *β∈*∈c*C* where the intersection pattern of *α* and *β* matches Rule 2 with respect to *α*_*i*_.

2. There is a *γ*∈*C* where the intersection pattern of *α* and *γ* matches Rule 2 with respect to *α*_*j*_.

##### 

Proof.(⇒) If *C* is pairwise incompatible, then by Corollary 1, there is a pair *α*,*β *∈ *C* whose intersection graph contains a cycle. Since the intersection graph is bipartite, this cycle must have length at least four and contain at least two states of each character. Let *α*_*i*_ and *α*_*j*_ be the two states of *α* on this cycle. Then, the intersection pattern of *α* and *β* matches Rule 2 with respect to both *α*_*i*_ and *α*_*j*_, and so the theorem holds. So we may assume that *C* is incompatible but pairwise compatible.

It follows from Corollary 3 that there exists an *α*∈*C* such that $Q_{\alpha }^{C}=\emptyset$. Then there must exist a character *β∈*∈c*C* such that the intersection pattern of *α* and *β* matches Rule 2 with respect to some state *α*_*i*_ of *α*; otherwise $S^{*}\in Q_{\alpha }^{C}$. Hence, $Q_{\alpha }^{C}\subseteq \{P^{i}\}$. Then, since $Q_{\alpha }^{C}=\emptyset$, there must be a character *γ*∈*C* such that the intersection pattern of *α* and *γ* places a constraint on $Q_{\alpha }^{C}$ that prevents $Q_{\alpha }^{C}$ from containing *P*^*i*^. There are two possibilities.

*Case 1*: There is a state *α*_*j*_ of *α* where *j*≠*i* and the intersection pattern of *α* and *γ* matches Rule 2 with respect to *α*_*j*_. In this case the theorem holds.

*Case 2*: The intersection pattern of *α* and *γ* matches Rule 1 with respect to *α*_*i*_. W.l.o.g., we fix *i*=1, and relabel the states of *α*, *β*, and *γ* so that *α*_1_∩*β*_1_≠*∅*, *α*_1_∩*β*_2_≠*∅*, *α*_2_∩*β*_1_≠*∅*, *α*_3_∩*β*_2_≠*∅*, *α*_1_⊆*γ*_1_, *α*_2_∩*γ*_2_≠*∅*, and *α*_3_∩*γ*_2_≠*∅*. Such a labeling exists since, by assumption, *α* and *β* matches Rule 2 with respect to *α*_1_, and *α* and *γ* matches Rule 1 with respect to *α*_1_.

If *α*_2_∩*γ*_1_≠*∅*, then the intersection pattern of *α* and *γ* matches Rule 2 with respect to *α*_2_, in which case the theorem holds. If *α*_3_∩*γ*_1_≠*∅*, then the intersection pattern of *α* and *γ* matches Rule 2 with respect to *α*_3_, in which case the theorem holds. So we may assume hat *α*_1_=*γ*_1_. Now, since *α*_1_∩*β*_1_≠*∅*, *α*_1_∩*β*_2_≠*∅*, and *α*_1_=*γ*_1_, we have that both *β*_1_∩*γ*_1_≠*∅* and *β*_2_∩*γ*_2_≠*∅*.

*γ*_3_ must have a nonempty intersection with at least one state of *α*, and since *α*_1_=*γ*_1_, we have that *α*_1_∩*γ*_3_=*∅*. So *γ*_3_ has a nonempty intersection with either *α*_2_ or *α*_3_. Due to the symmetry of the intersection graph of *α* and *β*, we may assume, w.l.o.g., that *α*_3_∩*γ*_3_≠*∅*.

By assumption, *α*_2_∩*γ*_1_=*∅*, and if *α*_2_∩*γ*_3_≠*∅*, then the intersection graph of *α* and *β* contains a cycle, contradicting our assumption that *C* is pairwise compatible. So we may assume that *α*_2_⊂*γ*_2_. Then, since *β*_1_∩*α*_2_≠*∅*, we have that *β*_1_∩*γ*_2_≠*∅*.

Let *s*∈*α*_3_∩*β*_2_. Since, by assumption, *α*_3_∩*γ*_1_=*∅*, we have that either *s*∈*γ*_2_ or *s*∈*γ*_3_. However, if *s*∈*γ*_2_, then *β*_2_∩*γ*_2_≠*∅* and intersection graph of *β* and *γ* contains a cycle, contradicting our assumption that *C* is pairwise compatible. Hence *s*∈*γ*_3_ and *β*_2_∩*γ*_3_≠*∅*.

**Figure 4:**
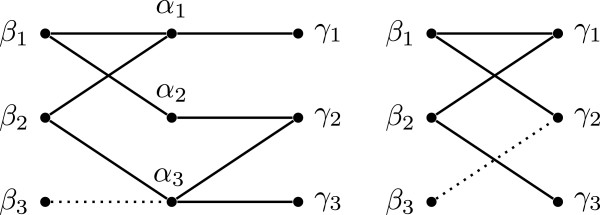
Illustrating the proof of Theorem 10.

We have now established all of the edges of the intersection graph of *α*, *β*, and *γ* represented by the solid edges in Figure 4. Now, let *s*_5_∈*α*_3_∩*γ*_2_. Now *s*_5_ must be in some state of *β*. If *s*_5_∈*β*_1_, then *s*_5_∈*β*_1_∩*α*_3_ and the intersection graph of *β* and *α* contains a cycle, contradicting our assumption that *C* is pairwise compatible. If *s*_5_∈*β*_2_, then *s*_5_∈*β*_2_∩*γ*_2_, and the intersection graph of *β* and *γ* contains a cycle, again contradicting our assumption that *C* is pairwise compatible. Hence *s*_5_∈*β*_3_. Then, we have that *s*_5_∈*β*_3_∩*α*_3_ and *s*_5_∈*β*_3_∩*γ*_2_, witnessing the dotted edges in Figure 4. So we have that the intersection pattern of *β* and *α* matches Rule 2 with *β*_2_ as witness, and the intersection pattern of *β* and *γ* matches Rule 2 with *β*_1_ as witness. Hence the theorem holds. □

Note that in the statement of Theorem 10, the characters *β* and *γ* are not necessarily distinct. In cases where they are not distinct, *C* contains an incompatible pair.

##### 

**Corollary 4.** A set *C* of 3-state characters is compatible if and only if every subset of at most three characters of *C* is compatible.

In [9], it was also shown that we can determine the compatibility of a pairwise compatible set *C* of three-state characters by testing the intersection patterns of *C* for the existence of one of a set of four forbidden patterns. As a corollary to Theorem 10, we have that a single forbidden pattern suffices to determine the compatibility of *C*.

##### 

**Figure 5:**
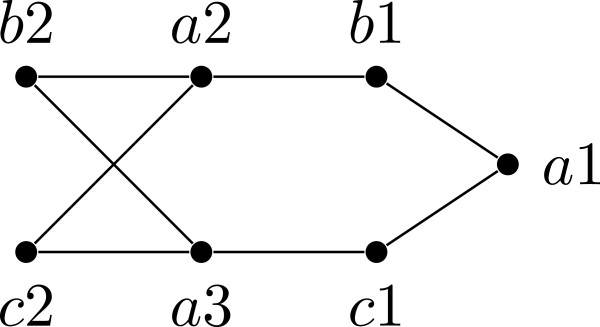
The forbidden subgraph for 3-state character compatibility.

**Corollary 5.** A pairwise compatible set *C* of 3-state characters is compatible if and only if the partition intersection graph of *C* does not contain, up to relabeling of characters and states, the subgraph of Figure 5.

Note that each edge of the graph of Figure 5 has one endpoint which is a state in *α*. It follows that we can find such a subgraph in the partition intersection graph of *C* by testing the intersection pattern of each pair of characters in *C*[10]. Furthermore, all *p* occurrences of the forbidden subgraph in the intersection graph of *m* characters on *n* taxa can be found in *O*(*m*^**2**^*n*+*p*) time. Whereas the forbidden subgraph given here is witnessed by eight taxa (or edges), each of the four forbidden subgraphs of [9] are witnessed by five taxa, making them better suited for taxon removal problems.

## Incompatible Triplets

**Figure 6:**
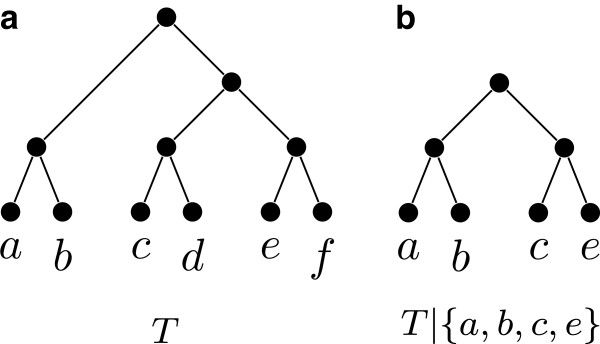
**Example of rooted phylogenetic trees.** (**a**) shows a tree *T* that is a witness that the triplets *ab*|*c*, *de*|*b*, *ef*|*c*, and *ec*|*b* are compatible; (**b**) shows the tree *T* restricted to the label set {*a*,*b*,*c*,*e*}.

A *rooted phylogenetic tree* (or just *rooted tree*) is a tree whose leaves are in one to one correspondence with a label set *L*(*T*), has a distinguished vertex called the *root*, and no vertex other than the root has degree two. See Figure 6(a) for an example. A rooted tree is *binary* if the root vertex has degree two, and every other internal (non-leaf) vertex has degree three. A *triplet* is a rooted binary tree with exactly three leaves. A triplet with label set {*a*,*b*,*c*} is denoted *ab*|*c* if the path between the leaves labeled *a* and *b* avoids the path between the leaf labeled *c* and the root vertex. For a tree *T*, and a label set *L*⊆*L*(*T*), let *T*^**′**^ be the minimal subtree of *T* connecting all the leaves with labels in *L*. The *restriction* of *T* to *L*, denoted by *T*|*L*, is the rooted tree obtained from *T*^**′**^ by distinguishing the vertex closest to the root of *T* as the root of *T*^**′**^, and suppressing every vertex other than the root having degree two. A rooted tree *Tdisplays* another rooted tree *T*^**′**^ if *T*^**′**^ can be obtained from *T*|*L*(*T*^**′**^) by contracting edges. A rooted tree *T* displays a collection of rooted trees T if *T* displays every tree in T. If such a tree *T* exists, then we say that T is compatible; otherwise, we say that T is incompatible. Given a collection of rooted trees T, it can be determined in polynomial time if T is compatible [3]**,**[25].

The following theorems follow from the connection between collections of unrooted trees with at least one common label across all the trees, and collections of rooted trees [3].

### 

**Theorem 11.** Let *Q* be a collection of quartets where every quartet in *Q* shares a common label *ℓ*. Let *R* be the set of triplets such that there exists a triplet *ab*|*c* in *R* if and only if there exists a quartet *ab*|*cℓ* in *Q*. Then, *Q* is compatible if and only if *R* is compatible.

Let *R* be a collection of triplets. For a subset *S*⊆*L*(*R*), we define the graph [*R*,*S*] as the graph having a vertex for each label in *S*, and an edge {*a*,*b*} if and only if *ab*|*c*∈*R* for some *c*∈*S*. The following theorem is from page 439 of [26].

### 

**Theorem 12.** A collection *R* of rooted triplets is compatible if and only if [*R*,*S*] is not connected for every *S*⊆*L*(*R*) with |*S*|≥3.

### 

**Corollary 6.** Let *R* be a set of rooted triplets such that *R* is incompatible but every proper subset of *R* is compatible. Then, [*R*,*L*(*R*)] is connected.

We now contrast our result on quartet compatibility with a result on triplets.

### 

**Theorem 13.** For every *n*≥3, if *R* is an incompatible set of triplets over *n* labels, and |*R*|>*n*−1, then some proper subset of *R* is incompatible.

### 

**Proof.** For sake of contradiction, let *R* be a set of triplets such that *R* is incompatible, every proper subset of *R* is compatible, |*L*(*R*)|=*n*, and |*R*|>*n*−1. The graph [*R*,*L*(*R*)] will contain *n* vertices and at least *n* edges. Since each triplet in *R* is distinct, there will be a cycle *C* of length at least three in [*R*,*L*(*R*)]. Since *R* is incompatible but every proper subset of *R* is compatible, by Corollary 6, [*R*,*L*(*R*)] is connected.

Consider any edge *e* in the cycle *C*. Let *t* be the triplet that contributed edge *e* in [*R*,*L*(*R*)]. Let *R*^**′**^**=***R*∖*t*. Since the graph [*R*,*L*(*R*)]−*e* is connected, [*R*^**′**^,*L*(*R*^**′**^)] is connected. By Theorem 12, *R*^**′**^ is incompatible. But *R*^**′**^**⊂***R*, contradicting that every proper subset of *R* is compatible. □

To show the bound is tight, we first prove a more restricted form of Theorem 2.

### 

**Theorem 14.** For every *n*≥4, there exists a set of quartets *Q* with |*L*(*Q*)|=*n*, and a label *ℓ*∈*L*(*Q*), such that all of the following hold. 

1. Every *q*∈*Q* contains a leaf labeled by *ℓ*.

2. *Q* is incompatible.

3. Every proper subset of *Q* is compatible.

4. |*Q*|=*n*−2.

### 

**Proof. **Consider the set of quartets *Q*_2,*n*−2_. From Lemmas 2 and 3, *Q*_2,*n*−2_ is incompatible but every proper subset of *Q*_2,*n*−2_ is compatible. The set *Q*_2,*n*−2_ contains exactly *n*−2 quartets. From the construction, there are two labels in *L* which are present in all the quartets in *Q*_2,*n*−2_. Set one of them to be *ℓ*. □

The following is a consequence of Theorems 14 and 11.

### 

**Corollary 7.** For every *n*≥3, there exists a set *R* of triplets with |*L*(*R*)|=*n* such that all of the following hold. 

1. *R* is incompatible.

2. Every proper subset of *R* is compatible.

3. |*R*|=*n*−1.

The generalization of the Fitch-Meacham examples given in [9] can also be expressed in terms of triplets. For any *r*≥2, let *L*={*a*,*b*_**1**_,*b*_**2**_,⋯,*b*_*r*_}. Let

$$R_{r}=ab_{r}|b_{1}\cup \overset{r-1}{\underset{i=1}{⋃}}ab_{i}|b_{i+1}$$

Let *Q*={*ab*|*cℓ*:*ab*|*c*∈*R*_*r*_} for some label *ℓ*∉*L*. The set *C*_*Q*_ of *r*-state characters corresponding to the quartet set *Q* is exactly the set of characters built for *r* in [9]. In the partition intersection graph of *C*_*Q*_, (following the terminology in [9]) labels *ℓ* and *a* correspond to the end cliques and the rest of the *r* labels {*b*_**1**_,*b*_**2**_,⋯,*b*_*r*_} correspond to the *r* tower cliques. From Lemma 5 and Theorem 11, *R*_*r*_ is compatible if and only of *Q* is compatible.

## Conclusion

We have shown that for every *r*≥2, $f(r)\geq \left\lfloor \frac{r}{2}\right\rfloor \cdot \left\lceil \frac{r}{2}\right\rceil +1$, by showing that for every *n*≥4, there exists an incompatible set *Q* of $\left\lfloor \frac{n-2}{2}\right\rfloor \cdot \left\lceil \frac{n-2}{2}\right\rceil +1$ quartets over a set of *n* labels such that every proper subset of *Q* is compatible. Previous results [1]**,**[6]**,**[9]**,**[13]**-**[15], along with our discussion in Section Incompatible Characters, show that our lower bound on *f*(*r*) is tight for *r*=2 and *r*=3. For quartets, our discussion in Section Incompatible quartets gives an upper bound on the maximum cardinality of a minimal set of incompatible quartets. However, this argument does not extend to multi-state characters. Indeed, an upper bound on the maximum cardinality of a minimal set of incompatible *r*-state characters remains a central open question. We give the following conjecture.

### 

**Conjecture 2. ***f*(*r*)∈*Θ*(*r*^**2**^).

A less ambituous goal would be to narrow the gap between the upper bound of *O*(*n*^**3**^) and lower bound of *Ω*(*n*^**2**^) on the maximum cardinality of a minimal incompatible set of quartets over *n* taxa given in Section Incompatible Quartets. Note that, due to Theorem 6, a proof of Conjecture 2 would also show that the number of incompatible quartets given in the statement of Theorem 2 is also as large as possible.

## Endnote

^**a**^**Rule 2 was state incorrectly in **[7].

## Competing interests

The authors declare that they have no competing interests.

## Authors’ contributions

BS was responsible for the lower bounds on character compatibility, the upper and lower bounds on quartet compatibility, the characterization of three-state character compatibility, and wrote all portions of the manuscript other than the section on triplet compatibility. SV was responsible for the upper and lower bounds on triplet compatibility, contributed to the lower bounds on quartet and character compatibility, and wrote the portion of the manuscript on triplet compatibility. DFB supervised the project. All authors read and approved the final manuscript.
